# Bis(3-hy­droxy­propanaminium) naphthalene-1,5-disulfonate

**DOI:** 10.1107/S1600536811053141

**Published:** 2011-12-21

**Authors:** Yu Jin

**Affiliations:** a Ordered Matter Science Research Center, Southeast University, Nanjing 211189, People’s Republic of China

## Abstract

In the title molecular salt, 2C_3_H_10_NO^+^·C_10_H_6_O_6_S_2_^2−^, the cations and anions are associated *via* N—H⋯O and O—H⋯O hydrogen-bonding inter­actions, giving rise to a three-dimensional structure with zigzag rows of cations lying between rows of anions. The asymmetric unit contains one cation and one half-anion, which is related to the remainder of the mol­ecule by an inversion center.

## Related literature

The title compound was studied as part of a search for simple ferroelectric compounds. For general background to ferroelectric metal-organic frameworks, see: Ye *et al.* (2006 ▶); Zhang *et al.* (2008 ▶, 2009 ▶, 2010 ▶); Fu *et al.* (2009 ▶).
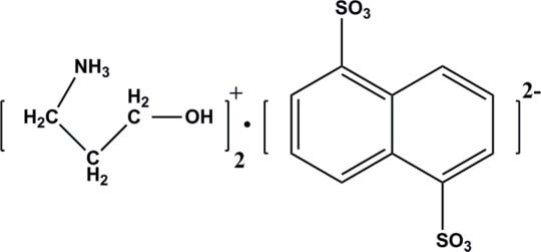


## Experimental

### 

#### Crystal data

2C_3_H_10_NO^+^·C_10_H_6_O_6_S_2_^2−^
*M* *_r_* = 438.51
Monoclinic, 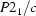

*a* = 10.004 (2) Å
*b* = 8.8311 (18) Å
*c* = 11.183 (2) Å
β = 92.79 (3)°
*V* = 986.8 (3) Å^3^
*Z* = 2
Mo *K*α radiation
μ = 0.32 mm^−1^
*T* = 293 K
0.3 × 0.3 × 0.2 mm

#### Data collection

Rigaku Mercury CCD diffractometer
Absorption correction: multi-scan (*CrystalClear*; Rigaku, 2005 ▶) *T*_min_ = 0.489, *T*_max_ = 1.000
9820 measured reflections
2268 independent reflections
2135 reflections with *I* > 2σ(*I*)
*R*_int_ = 0.029

#### Refinement

*R*[*F* ^2^ > 2σ(*F* ^2^)] = 0.054
*wR*(*F* ^2^) = 0.156
*S* = 1.08
2268 reflections
128 parameters
H-atom parameters constrained
Δρ_max_ = 0.58 e Å^−3^
Δρ_min_ = −0.63 e Å^−3^

### 

Data collection: *CrystalClear* (Rigaku, 2005 ▶); cell refinement: *CrystalClear*; data reduction: *CrystalClear*; program(s) used to solve structure: *SHELXS97* (Sheldrick, 2008 ▶); program(s) used to refine structure: *SHELXL97* (Sheldrick, 2008 ▶); molecular graphics: *SHELXTL* (Sheldrick, 2008 ▶); software used to prepare material for publication: *SHELXL97*.

## Supplementary Material

Crystal structure: contains datablock(s) I, global. DOI: 10.1107/S1600536811053141/ez2272sup1.cif

Structure factors: contains datablock(s) I. DOI: 10.1107/S1600536811053141/ez2272Isup2.hkl

Supplementary material file. DOI: 10.1107/S1600536811053141/ez2272Isup3.cml

Additional supplementary materials:  crystallographic information; 3D view; checkCIF report

## Figures and Tables

**Table 1:** Hydrogen-bond geometry (Å, °)

*D*—H⋯*A*	*D*—H	H⋯*A*	*D*⋯*A*	*D*—H⋯*A*
O4—H4*A*⋯O1^i^	0.82	1.95	2.772 (3)	177
N1—H1*D*⋯O3^ii^	0.89	1.93	2.768 (3)	157
N1—H1*C*⋯O4^iii^	0.89	2.07	2.854 (3)	147

Symmetry codes: (i) 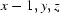; (ii) 
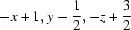
; (iii) 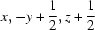.

**Fig. 1.**
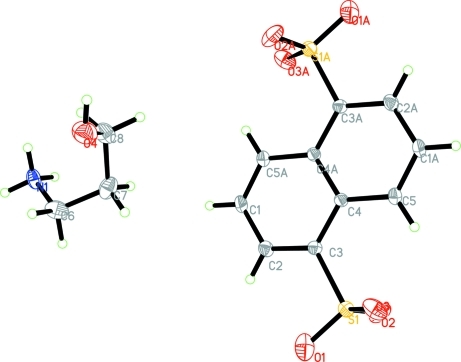
Crystal structure of the title compound with labelling and displacement ellipsoids drawn at the 30% probability level.

**Fig. 2.**
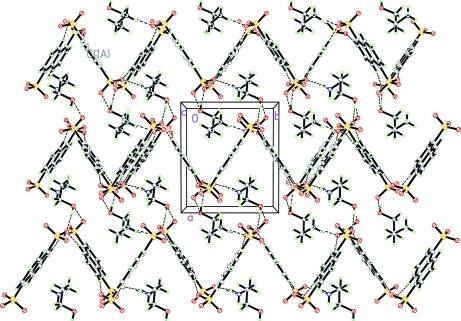
Crystal structure of the title compound with a view along the c axis. Intermolecular interactions are shown as dashed lines.

## supplementary crystallographic information

### Comment

Ferroelectric compounds have displayed such technical applications as ferroelectric random access memories (FeRAM), ferroelectric field-effect transistors, infrared detectors, piezoelectric sensors, nonlinear optical devices due to their excellent ferroelectric, piezoelectric, pyroelectric, and optical properties. A large number of new ferroelectric metal-organic coordination compounds corresponding to the necessary requirements for ferroelectric properties have been found, yet other necessary conditions, such as a phase transition, a good electric hysteresis loop and electric domain, and a dielectric anomaly, are often missed (Zhang *et al.*, 2009). Therefore pure organic compounds are of great potential and can make up for the drawbacks found in ferroelectric metal-organic coordination compounds. Reversible phase transitions remain one of the prominent properties for ferroelectrics. There exists a series of compounds in which the components can be arranged in a disordered fashion at a relatively high temperature and in an ordered fashion at a relatively low temperature and where the transition is reversible, which is called a reversible structual transition (Fu *et al.*, 2009; Zhang *et al.*, 2010; Zhang *et al.*, 2008; Ye *et al.*, 2006). The transition from the disordered arrangement to the ordered one leads to a sharp change in the physical properties of the compound. As part of our search for simple ferroelectric compounds I have investigated the title compound and report here its room temperature structure.

The centrosymmetric anion and one cation are shown in Fig. 1 with the hydrogen bonds listed in Table 1. The existence of numerous hydrogen-bonding interactions helps to make the substance more stable, so that it forms a three-dimensional layered structure. These interactions tie the cations and anions together in sheets with zigzag rows of cations lying between rows of anions (Fig. 2).

### Experimental

(C_3_H_10_NO)_2._(C_10_H_6_O_6_S_2_) was formed from a mixture of NH_2_(CH_2_)_3_OH (150.2 mg, 2.00 mmol), C_10_H_8_O_6_S_2_ (288.28 mg, 1.00 mmol), and distilled water (10 ml), which was stirred a few minutes at room temperature, giving a clear transparent solution. After evaporation for a few days, block colorless crystals suitable for X-ray diffraction were obtained in about 78% yield and filtered and washed with distilled water.

### Refinement

H atoms bound to carbon and nitrogen were placed at idealized positions [C—H = 0.93–0.97 Å, O—H = 0.82 Å and N—H = 0.89 Å] and allowed to ride on their parent atoms with *U*_iso_ fixed at 1.2 *U*_eq_(C,*N*).

### Crystal data

**Table:**

2C_3_H_10_NO^+^·C_10_H_6_O_6_S_2_^2^^−^	*F*(000) = 464
*M**_r_* = 438.51	*D*_x_ = 1.476 Mg m^−^^3^
Monoclinic, *P*2_1_/*c*	Mo *K*α radiation, λ = 0.71073 Å
*a* = 10.004 (2) Å	Cell parameters from 3450 reflections
*b* = 8.8311 (18) Å	θ = 6.2–55.3°
*c* = 11.183 (2) Å	µ = 0.32 mm^−^^1^
β = 92.79 (3)°	*T* = 293 K
*V* = 986.8 (3) Å^3^	Block, colorless
*Z* = 2	0.3 × 0.3 × 0.2 mm

### Data collection

**Table:**

Rigaku Mercury CCD diffractometer	2268 independent reflections
Radiation source: fine-focus sealed tube	2135 reflections with *I* > 2σ(*I*)
graphite	*R*_int_ = 0.029
ω scans	θ_max_ = 27.5°, θ_min_ = 3.1°
Absorption correction: multi-scan (*CrystalClear*; Rigaku, 2005)	*h* = −12→12
*T*_min_ = 0.489, *T*_max_ = 1.000	*k* = −11→11
9820 measured reflections	*l* = −14→14

### Refinement

**Table:**

Refinement on *F*^2^	Secondary atom site location: difference Fourier map
Least-squares matrix: full	Hydrogen site location: inferred from neighbouring sites
*R*[*F*^2^ > 2σ(*F*^2^)] = 0.054	H-atom parameters constrained
*wR*(*F*^2^) = 0.156	*w* = 1/[σ^2^(*F*_o_^2^) + (0.0777*P*)^2^ + 1.2249*P*] where *P* = (*F*_o_^2^ + 2*F*_c_^2^)/3
*S* = 1.08	(Δ/σ)_max_ < 0.001
2268 reflections	Δρ_max_ = 0.58 e Å^−^^3^
128 parameters	Δρ_min_ = −0.63 e Å^−^^3^
0 restraints	Extinction correction: *SHELXL97* (Sheldrick, 2008), Fc^*^=kFc[1+0.001xFc^2^λ^3^/sin(2θ)]^-1/4^
Primary atom site location: structure-invariant direct methods	Extinction coefficient: 0.254 (15)

### Special details

**Table:**

Geometry. All e.s.d.'s (except the e.s.d. in the dihedral angle between two l.s. planes) are estimated using the full covariance matrix. The cell e.s.d.'s are taken into account individually in the estimation of e.s.d.'s in distances, angles and torsion angles; correlations between e.s.d.'s in cell parameters are only used when they are defined by crystal symmetry. An approximate (isotropic) treatment of cell e.s.d.'s is used for estimating e.s.d.'s involving l.s. planes.
Refinement. Refinement of *F*^2^ against ALL reflections. The weighted *R*-factor *wR* and goodness of fit *S* are based on *F*^2^, conventional *R*-factors *R* are based on *F*, with *F* set to zero for negative *F*^2^. The threshold expression of *F*^2^ > σ(*F*^2^) is used only for calculating *R*-factors(gt) *etc*. and is not relevant to the choice of reflections for refinement. *R*-factors based on *F*^2^ are statistically about twice as large as those based on *F*, and *R*- factors based on ALL data will be even larger.

### Fractional atomic coordinates and isotropic or equivalent isotropic displacement parameters (Å^2^)

**Table:**

	*x*	*y*	*z*	*U*_iso_*/*U*_eq_	
C1	0.4862 (3)	−0.0066 (3)	0.7251 (2)	0.0316 (5)	
H1B	0.4639	−0.0242	0.8037	0.038*	
C2	0.5997 (2)	0.0809 (3)	0.7025 (2)	0.0293 (5)	
H2A	0.6513	0.1217	0.7660	0.035*	
C3	0.6347 (2)	0.1062 (2)	0.58760 (19)	0.0243 (5)	
C4	0.5578 (2)	0.0439 (2)	0.48811 (18)	0.0227 (5)	
C5	0.5915 (2)	0.0660 (3)	0.36695 (19)	0.0274 (5)	
H5A	0.6675	0.1216	0.3505	0.033*	
C6	0.2313 (3)	0.2177 (3)	1.0492 (3)	0.0468 (7)	
H6A	0.3213	0.2261	1.0849	0.056*	
H6B	0.1862	0.3135	1.0606	0.056*	
C7	0.2388 (3)	0.1877 (3)	0.9175 (3)	0.0444 (7)	
H7A	0.2867	0.0936	0.9068	0.053*	
H7B	0.2900	0.2681	0.8824	0.053*	
C8	0.1045 (3)	0.1771 (3)	0.8510 (3)	0.0455 (7)	
H8A	0.1151	0.1265	0.7750	0.055*	
H8B	0.0448	0.1163	0.8971	0.055*	
N1	0.1620 (2)	0.1016 (2)	1.10823 (18)	0.0337 (5)	
H1C	0.1596	0.1236	1.1858	0.051*	
H1D	0.2041	0.0138	1.0994	0.051*	
H1E	0.0789	0.0946	1.0766	0.051*	
O1	0.8140 (2)	0.2850 (3)	0.68268 (19)	0.0555 (7)	
O2	0.8824 (2)	0.1062 (2)	0.5327 (2)	0.0544 (6)	
O3	0.7522 (2)	0.3249 (2)	0.4755 (2)	0.0477 (6)	
O4	0.0430 (3)	0.3286 (3)	0.8284 (2)	0.0597 (7)	
H4A	−0.0252	0.3192	0.7853	0.090*	
S1	0.78281 (5)	0.21407 (7)	0.56780 (5)	0.0287 (3)	

### Atomic displacement parameters (Å^2^)

**Table:**

	*U*^11^	*U*^22^	*U*^33^	*U*^12^	*U*^13^	*U*^23^
C1	0.0386 (13)	0.0368 (12)	0.0198 (10)	−0.0004 (10)	0.0053 (8)	0.0012 (9)
C2	0.0342 (12)	0.0317 (11)	0.0217 (10)	−0.0001 (9)	−0.0020 (8)	−0.0042 (8)
C3	0.0247 (10)	0.0233 (10)	0.0248 (10)	0.0014 (8)	−0.0003 (8)	0.0001 (8)
C4	0.0259 (10)	0.0206 (9)	0.0217 (10)	0.0033 (8)	0.0013 (8)	−0.0001 (7)
C5	0.0304 (11)	0.0281 (11)	0.0241 (10)	−0.0018 (8)	0.0051 (8)	0.0018 (8)
C6	0.0530 (17)	0.0347 (14)	0.0515 (17)	−0.0034 (12)	−0.0110 (14)	0.0036 (12)
C7	0.0441 (15)	0.0363 (14)	0.0541 (17)	0.0039 (11)	0.0158 (13)	0.0092 (12)
C8	0.0631 (19)	0.0346 (14)	0.0389 (14)	−0.0114 (13)	0.0047 (13)	−0.0003 (11)
N1	0.0504 (12)	0.0239 (9)	0.0275 (10)	0.0065 (9)	0.0082 (9)	−0.0002 (7)
O1	0.0463 (12)	0.0836 (17)	0.0363 (11)	−0.0292 (11)	−0.0021 (9)	−0.0123 (10)
O2	0.0376 (11)	0.0419 (11)	0.0858 (17)	0.0065 (9)	0.0251 (11)	0.0102 (11)
O3	0.0583 (13)	0.0309 (10)	0.0524 (12)	−0.0130 (9)	−0.0124 (10)	0.0137 (9)
O4	0.0562 (14)	0.0665 (15)	0.0552 (13)	0.0032 (12)	−0.0102 (10)	−0.0073 (12)
S1	0.0277 (4)	0.0296 (4)	0.0285 (4)	−0.0031 (2)	−0.0004 (2)	0.0019 (2)

### Geometric parameters (Å, °)

**Table:**

C1—C5^i^	1.365 (3)	C6—H6B	0.9700
C1—C2	1.407 (3)	C7—C8	1.507 (5)
C1—H1B	0.9300	C7—H7A	0.9700
C2—C3	1.366 (3)	C7—H7B	0.9700
C2—H2A	0.9300	C8—O4	1.489 (4)
C3—C4	1.432 (3)	C8—H8A	0.9700
C3—S1	1.784 (2)	C8—H8B	0.9700
C4—C5	1.426 (3)	N1—H1C	0.8900
C4—C4^i^	1.428 (4)	N1—H1D	0.8900
C5—C1^i^	1.365 (3)	N1—H1E	0.8900
C5—H5A	0.9300	O1—S1	1.450 (2)
C6—N1	1.418 (4)	O2—S1	1.446 (2)
C6—C7	1.502 (4)	O3—S1	1.445 (2)
C6—H6A	0.9700	O4—H4A	0.8200
			
C5^i^—C1—C2	120.7 (2)	C8—C7—H7A	108.7
C5^i^—C1—H1B	119.7	C6—C7—H7B	108.7
C2—C1—H1B	119.7	C8—C7—H7B	108.7
C3—C2—C1	120.3 (2)	H7A—C7—H7B	107.6
C3—C2—H2A	119.8	O4—C8—C7	112.3 (2)
C1—C2—H2A	119.8	O4—C8—H8A	109.1
C2—C3—C4	121.0 (2)	C7—C8—H8A	109.1
C2—C3—S1	117.15 (17)	O4—C8—H8B	109.1
C4—C3—S1	121.80 (16)	C7—C8—H8B	109.1
C5—C4—C4^i^	118.9 (2)	H8A—C8—H8B	107.9
C5—C4—C3	122.9 (2)	C6—N1—H1C	109.5
C4^i^—C4—C3	118.3 (2)	C6—N1—H1D	109.5
C1^i^—C5—C4	120.8 (2)	H1C—N1—H1D	109.5
C1^i^—C5—H5A	119.6	C6—N1—H1E	109.5
C4—C5—H5A	119.6	H1C—N1—H1E	109.5
N1—C6—C7	112.2 (2)	H1D—N1—H1E	109.5
N1—C6—H6A	109.2	C8—O4—H4A	109.5
C7—C6—H6A	109.2	O3—S1—O2	112.18 (15)
N1—C6—H6B	109.2	O3—S1—O1	111.71 (15)
C7—C6—H6B	109.2	O2—S1—O1	113.79 (16)
H6A—C6—H6B	107.9	O3—S1—C3	107.56 (12)
C6—C7—C8	114.2 (3)	O2—S1—C3	105.61 (12)
C6—C7—H7A	108.7	O1—S1—C3	105.37 (11)

Symmetry codes: (i) −*x*+1, −*y*, −*z*+1.

### Hydrogen-bond geometry (Å, °)

**Table:**

*D*—H···*A*	*D*—H	H···*A*	*D*···*A*	*D*—H···*A*
O4—H4A···O1^ii^	0.82	1.95	2.772 (3)	177.
N1—H1D···O3^iii^	0.89	1.93	2.768 (3)	157.
N1—H1C···O4^iv^	0.89	2.07	2.854 (3)	147.

Symmetry codes: (ii) *x*−1, *y*, *z*; (iii) −*x*+1, *y*−1/2, −*z*+3/2; (iv) *x*, −*y*+1/2, *z*+1/2.

## References

[bb1] Fu, D.-W., Ge, J.-Z., Dai, J., Ye, H.-Y. & Qu, Z.-R. (2009). *Inorg. Chem. Commun.* **12**, 994–997.

[bb2] Rigaku (2005). *CrystalClear* Rigaku Corporation, Tokyo, Japan.

[bb3] Sheldrick, G. M. (2008). *Acta Cryst.* A**64**, 112–122. 10.1107/S0108767307043930 18156677

[bb4] Ye, Q., Song, Y.-M., Wang, G.-X., Chen, K. & Fu, D.-W. (2006). *J. Am. Chem. Soc.* **128**, 6554–6555. 10.1021/ja060856p 16704244

[bb5] Zhang, W., Li, Z.-C., Xiong, R.-G., Nakamura, T. & Huang, S.-P. (2009). *J. Am. Chem. Soc.* **131**, 12544–12545. 10.1021/ja905399x 19685869

[bb6] Zhang, W., Xiong, R.-G. & Huang, S.-P. D. (2008). *J. Am. Chem. Soc.* **130**, 10468–10469. 10.1021/ja803021v 18636707

[bb7] Zhang, W., Ye, H.-Y., Cai, H.-L., Ge, J.-Z. & Xiong, R.-G. (2010). *J. Am. Chem. Soc.* **132**, 7300–7302. 10.1021/ja102573h 20459097

